# Cell polarity changes in cancer initiation and progression

**DOI:** 10.1083/jcb.202308069

**Published:** 2023-12-13

**Authors:** Florent Peglion, Sandrine Etienne-Manneville

**Affiliations:** 1 Cell Polarity, Migration and Cancer Unit, Université de Paris, UMR3691 CNRS, Equipe Labellisée Ligue 2023, Institut Pasteur, Paris, France

## Abstract

Cell polarity, which consists of the morphological, structural, and functional organization of cells along a defined axis, is a feature of healthy cells and tissues. In contrast, abnormal polarity is a hallmark of cancer cells. At the molecular level, key evolutionarily conserved proteins that control polarity establishment and maintenance in various contexts are frequently altered in cancer, but the relevance of these molecular alterations in the oncogenic processes is not always clear. Here, we summarize the recent findings, shedding new light on the involvement of polarity players in cancer development, and discuss the possibility of harnessing cell polarity changes to better predict, diagnose, and cure cancers.

## Teaser

Peglion and Etienne-Manneville discuss new insights into the pleiotropic effects of cell polarity alteration in cancer pathophysiology.

## Introduction

The control of cell polarity relies on a set of evolutionary conserved proteins—so-called polarity proteins—that self-organize into dynamic complexes (Peglion and Goehring, 2019). These polarity complexes set up the three-dimensional organization of the cell by defining the apical and basolateral domains of epithelial cells (basoapical polarity; Roignot et al., 2013), the front and the rear of migrating cells (Etienne-Manneville, 2008), the immunological synapse in T-cell (Angus and Griffiths, 2013), or the axon and dendrites of neurons (Bentley and Banker, 2016). They also dictate mitotic spindle orientation and affect the cell fate determinants’ segregation during asymmetric cell division (ACD). Therefore, alterations of cell polarity are associated with dramatic morphological and functional changes at the cell and tissue levels and have been suggested to play a key role in cancer formation and progression.

Since the discovery that PAR genes ensure cell fate determinants partitioning during the asymmetrical division of *Caenorhabditis elegans* zygotes (Kemphues et al., 1988), decades of research have characterized a web of polarity proteins, which control polarity in a wide variety of cellular contexts. For simplicity, three main polarity protein complexes exist: (1) the PAR network, composed of cell cortex–enriched PAR6/aPKC/PAR3 and PAR1 and cytoplasmic PAR-4 (LKB1) and PAR-5 (14-3-3 protein family), (2) the Crumbs/Pals1/PATJ complex, and (3) the SCRIB/LGL/DLG complex. In addition, the Rho-GTPases Cdc42, Rac1, and RhoA along with the phosphoinositides PI(3,4,5)P3 and PI(4,5)P2 and their regulators PTEN and PI3K play a crucial role in cell polarity by regulating the activity of these polarity complexes (Zhan et al., 2008). Finally, many proteins involved in cell–cell adhesion, like cadherins, or in cell–matrix adhesions, like integrins, influence polarity establishment and maintenance, and are frequently altered in tumors (Janiszewska et al., 2020). In cell collectives, a larger scaler polarization, called planar polarity, organizes the cellular positions and functions within a tissue, in particular within the epithelium. We will refer the reader eager for molecular details of cell and tissue polarization to the excellent reviews on basoapical polarity (Buckley and St Johnston, 2022; Flores-Benitez and Knust, 2016) and planar cell polarity (Butler and Wallingford, 2017). This review will mainly focus on the most recent studies examining how cell polarity changes contribute to cancer initiation and progression. Due to space limitation, the implication of planar polarity will not be discussed here, although some planar polarity proteins have also been involved in cancer progression (Pastushenko et al., 2021; VanderVorst et al., 2018). Also, because 80–90% of human cancers are carcinomas emerging from transformed epithelial cells, we will more specifically explore the role of basoapical epithelial cell polarity alteration in the initiation and the invasive progression of carcinomas. Finally, the more unexpected impact of polarity protein alterations on tumor therapeutics resistance will be discussed.

### Alteration of epithelial polarity during carcinogenesis

Although the loss of polarity is described as a hallmark of cancer, most cancer cells show an altered basoapical and/or planar polarity replaced by a different kind of polarized organization fitting their new functions such as cell migration and invasion. These profound modifications of cell polarity are observed in most carcinomas (Almagro et al., 2022) and are often linked to direct alterations of polarity proteins’ expression (Huang and Muthuswamy, 2010; Lin et al., 2015; Saito et al., 2018). For instance, in 40% of 432 invasive breast cancer tissues from heterogeneous molecular subtypes in which all cells have lost their basoapical polarity, the copy number of genes coding for core polarity proteins is altered (Catterall et al., 2020). The increased presence of unpolarized cell clones as tumor switches from pre- to a fully invasive nature also highlights the selective advantage associated with polarity alteration (Halaoui et al., 2017). Although evidence showing that carcinogenic activation and cell transformation affect cell polarity to promote tissue disruption and tumor progression is accumulating (Aranda et al., 2006; Gardiol et al., 1999; Messal et al., 2019; Nakagawa et al., 2000; Pastushenko et al., 2021), the direct role of cell polarity alterations in carcinogenesis remains disputed. Here, we will focus on studies investigating the direct role of cell polarity alterations in cancer initiation and progression.

The alterations of cell polarity in tumors can be classified into two main categories depending on whether the alterations in polarity proteins (molecular changes) have proven consequences on downstream cellular polarity (phenotypic changes). Recent reviews have detailed the occurrence in human tumors of frequent alterations of genes involved in the basoapical polarity (Atashrazm and Ellis, 2021; Reina-Campos et al., 2019), front-rear polarity (Gandalovicova et al., 2016), or T-cell polarity (Mastrogiovanni et al., 2022a). These molecular changes include (1) gene repression (deletion and loss of function mutations or transcriptional downregulation) such as *lkb1* and *pten* in gastrointestinal hamartomatous polyposis syndromes (Boland et al., 2022), or *prkci/z* in aggressive colorectal cancers (Nakanishi et al., 2018), or (2) gene overexpression (from gene amplification or transcriptional upregulation), such as *lgl2* in breast cancer positive for estrogen receptors (Saito et al., 2019). Moreover, at the protein level, the mislocalization of polarity protein is another critical alteration frequently leading to polarity defects during mammary (Zhan et al., 2008) and prostate tumorigenesis (Pearson et al., 2011). Finally, the proteolytic degradation of polarity proteins by oncogenic viruses participates directly in cancer initiation (Gardiol et al., 1999; Nakagawa et al., 2000).

Phenotypically, tumors bear the complete loss of cell polarity (Catterall et al., 2020), intermediate polarity loss (Halaoui et al., 2017), or inverse polarity (Canet-Jourdan et al., 2022; Halaoui et al., 2017; Zajac et al., 2018). However, these changes can be transient with cancer cells displaying functional polarization during collective invasion and circulation in the vasculature (Heikenwalder and Lorentzen, 2019; Lorentzen et al., 2018). Finally, identical polarity protein change can have pro or antitumoral effects depending on the tumor type (Iden et al., 2012; Reina-Campos et al., 2019). The specific characteristics of each type of epithelium may explain the discrepancy in phenotypes of single polarity protein depletion in various tissues, even within the same species.

### Changes in cell polarity contribute to tissue disorganization and cancer initiation

Cell polarity determines the position and orientation of the cell division plane and the asymmetric distribution of cell fate determinants. Thus, it is essential in the control of tissue organization. In the context of epithelial tissue, the fine-tuned balance between planar-symmetric and asymmetric cell division controls tissue homeostasis (Bryant and Mostov, 2008). In general, planar divisions are proliferative and increase the stem cell pool. In contrast, divisions perpendicular to the epithelial sheet, coined non-planar divisions or asymmetric cell divisions (ACD), promote the differentiation of one of the daughter cells and tissue stratification (see glossary text box). Thus, the control of the orientation of cell divisions is essential for the tissue homeostatic program in stratified epithelium like the epidermis or the neuroepithelium (Lechler and Fuchs, 2005; Seldin et al., 2016). Perturbation of this regulation can trigger hyperproliferation and multilayering, expansion toward the lumen, and disruption of the basal membrane, the three phenomena associated with the early stage of carcinogenesis. An increased proportion of ACD can trigger the accumulation of abnormally positioned cells at the basal side, while inhibition of ACD can promote overgrowth because of increased planar, proliferative division (Lechler and Mapelli, 2021; Niessen et al., 2012).

**Figure 1.**
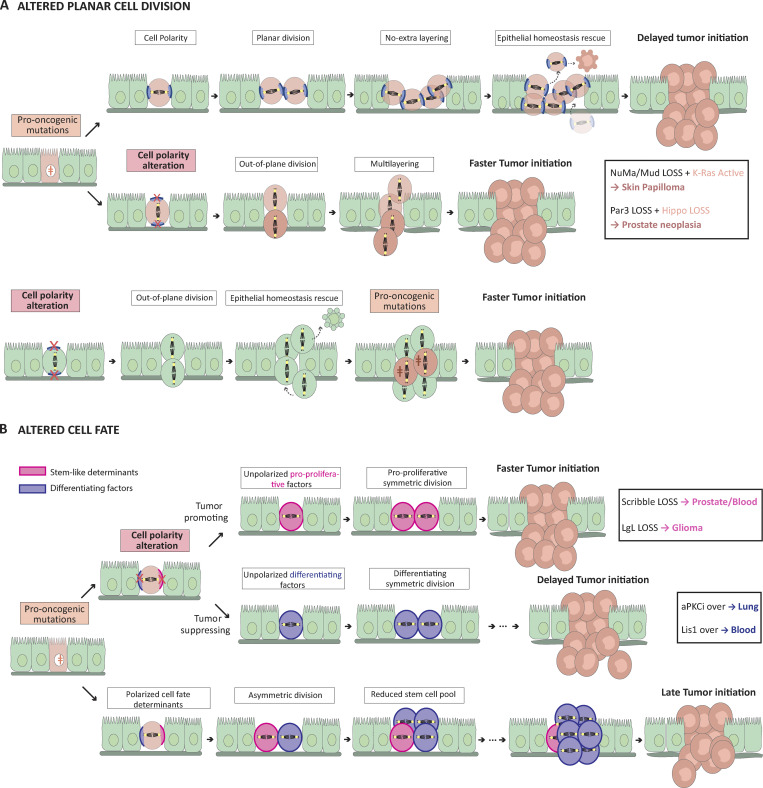
**Altered cell polarity affects cancer initiation. (A)** Changes in epithelial progenitor cell polarity can lead to mitotic spindle complex (Gαi-LGN-NuMA, in blue) misalignment and non-planar cell division. Whether these changes occur before or after pro-oncogenic mutations, they hasten the initiation of a tumor by promoting epithelial multilayering and tissue architecture alteration. Epithelial homeostasis rescue is a mechanism limiting the potential oncogenic impact of such tissue disorganization. It includes cell extrusion from the epithelial layer and apoptosis initiation (Lechler and Mapelli, 2021). Epithelial homeostasis rescue fails when tissue architecture alteration becomes too severe, typically after both cell polarity changes and oncogenic mutations. **(B)** Carcinoma stem cell polarity alterations can affect the cell fate of its progeny, by preventing the asymmetrical segregation of cell fate determinants which normally delays cancer initiation and growth. If it results in equal segregation of pro-proliferating determinants (pink) and the generation of two proliferative cancer cells, the polarity change will promote tumor formation. On the other hand, in some cancers, the asymmetric-to-symmetric segregation of differentiating factors (blue) leads to the generation of two differentiating cells and delays cancer initiation.

Alteration of spindle positioning increasing out-of-plane divisions is associated with pro-oncogenic multilayering of monolayered epithelium (Fig. 1 A). An early study in *Drosophila* wing disc epithelium highlighted that Dlg1 or Scrib alteration induces basal cell delamination, but it is not sufficient to induce tumor formation (Nakajima et al., 2013). However, blocking apoptosis of delaminated cells then leads to dramatic tissue neoplasia with invasive characteristics. The correct orientation of the mitotic spindle depends on coupling the astral microtubules-interacting spindle complex (Gαi/LGN/NuMA/dynein) with the 3D epithelium cues such as cell–cell and cell–matrix adhesion cell polarity proteins (Lechler and Mapelli, 2021; Segalen and Bellaïche, 2009; Vorhagen and Niessen, 2014). Ablation of the spindle complex protein NuMA in the mammalian epidermis only leads to tumor formation upon additional k-RAS oncogenic insult (Morrow et al., 2019). Removal of Par3 in both luminal and basal prostatic epithelial cells changes the orientation of the mitotic spindle, leading to multilayered epithelial premalignant structures called high-grade intraepithelial neoplasia (PIN). This Par3-phenotype requires Hippo pathway attenuation. Complete inhibition of Hippo by the codeletion of Lats-1 in Par3-deleted prostatic cells leads to the development of adenocarcinoma with local microinvasion phenotype, a much more severe type of prostate cancer. A higher proportion of misorientated spindles in the double KO cells suggests that a robust alteration of the division plane may participate in the initiation of this type of cancer (Zhou et al., 2019). Finally, breast cancer progression is associated with progressive loss of basoapical polarity in human and mouse models. The multilayering of the mammary duct epithelium is associated with the misorientation of mitotic spindle and basal delamination (Halaoui et al., 2017). However, oblique divisions in normal duct epithelium are not sufficient to induce cell delamination and stratification (Halaoui et al., 2017). Overall, out-of-plane cell divisions do not appear sufficient to trigger tumor formation, but they seem to promote tumor initiation when combined with oncogenic alterations.

Another important consequence of spindle misalignment during ACD is the altered segregation of cell fate determinants (Fig. 1 B). Interfering with cell fate determinants can promote the emergence of neoplasia-like characteristics (e.g., centrosome duplication and genomic instability; Caussinus and Gonzalez, 2005). Genetic alteration of the cell fate determinants Numb and Numb-like in mammalian forebrain neural cell progenitor causes hyperproliferation and lack of differentiation (Klezovitch et al., 2004; Li et al., 2003). In *Drosophila* neuroblasts, mutations in the Scrib complex (Gateff, 1978) or the spindle positioning complex (Caussinus and Gonzalez, 2005) prevent ACD and trigger hyperproliferative neuroblastomas-like tumors. In mice, conditional ablation of Scrib increases cell renewal capacities, induces hyperplasia, and predisposes the prostate and blood tissues to tumorigenesis (Elsum et al., 2014; Godde et al., 2014; Mohr et al., 2018). Hints at the molecular mechanisms have been uncovered in hematopoietic stem cells. There, Scrib loss alters the activation of the PAR complex member, Cdc42, and the polarization of the Hippo pathway. The asymmetric segregation of myc is lost and symmetric cell division and self-renewal capacity of the hematopoietic stem cell pool are increased (Althoff et al., 2020). Interestingly, decreased ACD in the oligodendrocytes progenitor cells (OPC) is observed in premalignant stages of human oligodendrogliomas and can trigger tumor initiation in a mouse model of the disease (Sugiarto et al., 2011). Mechanistically, removing Lgl1 from the OPC prevents the asymmetric segregation of the fate determinant NG2 to increase proliferative symmetric cell divisions and facilitate gliomagenesis (Daynac et al., 2018). Alternatively, the specific depletion of Par3 in neural precursor cells triggers telencephalon hypertrophy due to altered cleavage plane orientation and increased proliferative divisions during the neurogenesis phase (Hirose et al., 2022; Liu et al., 2018). The importance of Par3 in controlling the segregation of differentiating factors and subsequently the proliferative index of stem cells was also observed in the intestinal tissue. Using lineage tracing in vivo, Wu and colleagues revealed the existence of a Par3-dependent threshold of the differentiating factor Pros to maintain a predetermined number of divisions in intestinal stem cells (Wu et al., 2023). These data are in favor of a direct and causal link between altered cell fate segregation and the promotion of cancer initiation. However, one should also note that alteration of ACD can, in some cases, limit tumor development (Pine et al., 2010). For instance, alteration of the dynein binding protein Lis-1, which controls spindle positioning (Schwamborn and Knoblich, 2008), blocks the progression of myeloid leukemia by accelerating differentiation through defects in cell fate determinant inheritance (Zimdahl et al., 2014). In lung adenocarcinoma initiating cells, the depletion of aPKCι, another member of the PAR complex, reduces the cell asymmetrical division, strongly impairs tumor initiation, and increases mice survival when cells are injected orthotopically in mouse lungs (Ali et al., 2016). In the hematopoietic system, human BCR-ABL+ leukemic B-cell progenitor cells show an overactivation of aPKCι and an upregulation of Cdc42 (Nayak et al., 2019). Serial transplantation of aPKCι-depleted leukemic progenitor cells prevents the death of mice while control mice die of acute B lymphoblastic leukemia (B-ALL). aPKCι-depletion restores B-cell differentiation, partly by reducing cell fate determinant Numb cytoplasmic mislocalization (Nayak et al., 2019).

The impact of cell polarity perturbations on tissue organization and tumor initiation involves not only the alteration of the cell division axis but also alterations of other polarized cell behaviors such as cell extrusion or cell migration. Poor-prognosis-associated aPKCι ovexpression in breast cancer induces basal extrusion into the myoepithelium of the mammary gland (Awadelkarim et al., 2012; Villeneuve et al., 2019). The increased aPKCι alters cell–cell adhesions between transformed and untransformed cells to promote basal extrusion. Whether altered intercellular adhesion also affects planar cell division to increase epithelial stratification has not been investigated.

Overall, these studies highlight that polarity-dependent alteration of tissue homeostasis can induce tumors only on rare occasions. The polarity-dependent defects are likely buffered by safeguarding mechanisms. These results suggest that early cell polarity alterations are tumor permissive rather than bona fide tumor promoters. However, key polarity proteins have additional signaling roles that directly impact cell proliferation, survival, apoptosis, and quiescence. The fact that safeguard mechanisms, like apoptosis, are controlled by cell polarity proteins (Zhan et al., 2008) potentially explains why, in some cases, specific changes in polarity are sufficient to induce tumor formation.

### Alteration of cell polarity promotes tumor growth

While the impact of alterations of cell polarity on tissue organization was expected, their direct consequence in the regulation of cell and tumor growth has emerged only recently (Fomicheva et al., 2020). In general, polarity proteins are involved in multiple pathways, including the Hippo, mTOR, Hedgehog (HH), JAK/STAT, or MAPK pathways, which influence cell proliferation. This is well exemplified in *Drosophila*, in which *scrib*- and *dlg*-mutant epithelial cells upregulate promitotic JAK/STAT activity during tumor initiation (Bunker et al., 2015). In polarity-deficient epithelial cells, the abnormal activity of aPKC also fuels tumor overgrowth by hyperactivating the Hippo pathway (Bunker et al., 2015). However, since the Hippo pathway also controls spindle orientation (Keder et al., 2015), its alteration may also promote cell proliferation by affecting the balance between asymmetric and symmetric division.

Alteration of Scrib activates other key protumorigenic pathways. The mislocalization of Scrib disrupts PTEN location and activates the Akt/mTOR/S6K pathway in basal breast cancer (Feigin et al., 2014). In prostate tissue, the deletion of Scrib promotes tumorigenesis by activation of the pro-proliferative MAPK pathway (Pearson et al., 2011), while it promotes Myc-dependent breast tumor initiation by reducing myc-induced apoptosis (Zhan et al., 2008). Similarly, the polarity protein Par3 controls cell proliferation in multiple ways (Lv et al., 2022). In the mammalian brain, the loss of Par3 promotes Hippo pathway activation in radial glia progenitors and leads to massive cortex enlargement and heterotopia (Liu et al., 2018). Selective deletion of Par3 in the telencephalon leads to the hyperactivation of the pro-oncogenic Hedgehog (HH) signaling, which alters the brain tissue architecture during neurogenesis (Hirose et al., 2022). Par3 acts together with aPKCι to sustain ERK-, AKT-, and NF-kB/Stat-3-dependent pro-proliferation activity during the initiation of chemically induced skin carcinogenesis (Vorhagen et al., 2018). In BCC, overexpressed aPKCι phosphorylates Gli1 and increases its DNA-binding affinity to promote HH target gene expression, including the gene coding for aPKCi (Atwood et al., 2013). aPKCi also affects Gli1 acetylation, which is critical for Gli1 nuclear translocation and full activation (Mirza et al., 2019). Par3 also influences cell proliferation and survival in different and sometimes opposing ways in the same tissue. Deletion of Par3 in a chemically induced model of carcinogenesis involving Ras mutations favors the appearance of the rare keratoancathomas (KA) tumor type but reduces the formation of skin papillomas (Iden et al., 2012). The opposite functions of Par3 in these two tumors likely reflect differences in its localization. In skin-papillomas, Par3 is enriched at cell–cell junctions, where it recruits aPKC to activate the Ras-MAPK. Par3 loss mislocalizes aPKC and Ras in the cytoplasm which reduces MAPK pathway activity, cell proliferation, and survival. In contrast, in KA, Par3 is not present at cell junctions but localizes in cytoplasm. Here, the depletion of Par3 results in an increased activity of CRaf, leading to cell proliferation (Iden et al., 2012). Par3 tumor-promoting activity in the epidermis also protects genome integrity, mitotic fidelity, and premature skin cell differentiation (Dias Gomes et al., 2019).

**Glossary**

**Asymmetric Cell Division (ACD):** Cell division resulting in daughter cells bearing different cell fates. This can be the result of asymmetric cell fate determinants segregation during mitosis, geometrical asymmetric division, or non-planar division (see below) leading to daughter cells being exposed to different cues affecting their cell fate.

**Planar division:** Cell division leading to the two daughter cells remaining in the plane of the tissue. It is the result of the mitotic spindle being positioned parallel to the plane of the tissue. The planar division usually leads to daughter cells keeping their proliferative identity.

**Non-planar division:** Cell division leading to a daughter cell being produced out of the tissue. It results from the mitotic spindle being abnormally aligned perpendicularly to the plane of the tissue.

The extra-layered daughter cell usually differentiates whereas the cell remaining in the tissue remains proliferative. By extension, the non-planar division is often referred to as being an asymmetric cell division (Fig. 1). Readers eager for more details are referred to Lechler and Mapelli (2021).

**Extracellular matrix (ECM):** Network of intercellular macromolecules composed of collagens, elastin, and glycoproteins. ECM plays a critical role in establishing and maintaining cell polarity mostly through engagement with transmembrane adhesion receptors such as integrins which transmit to the cell the biophysical properties of its environment (Tharp and Weaver, 2018).

**Figure 2.**
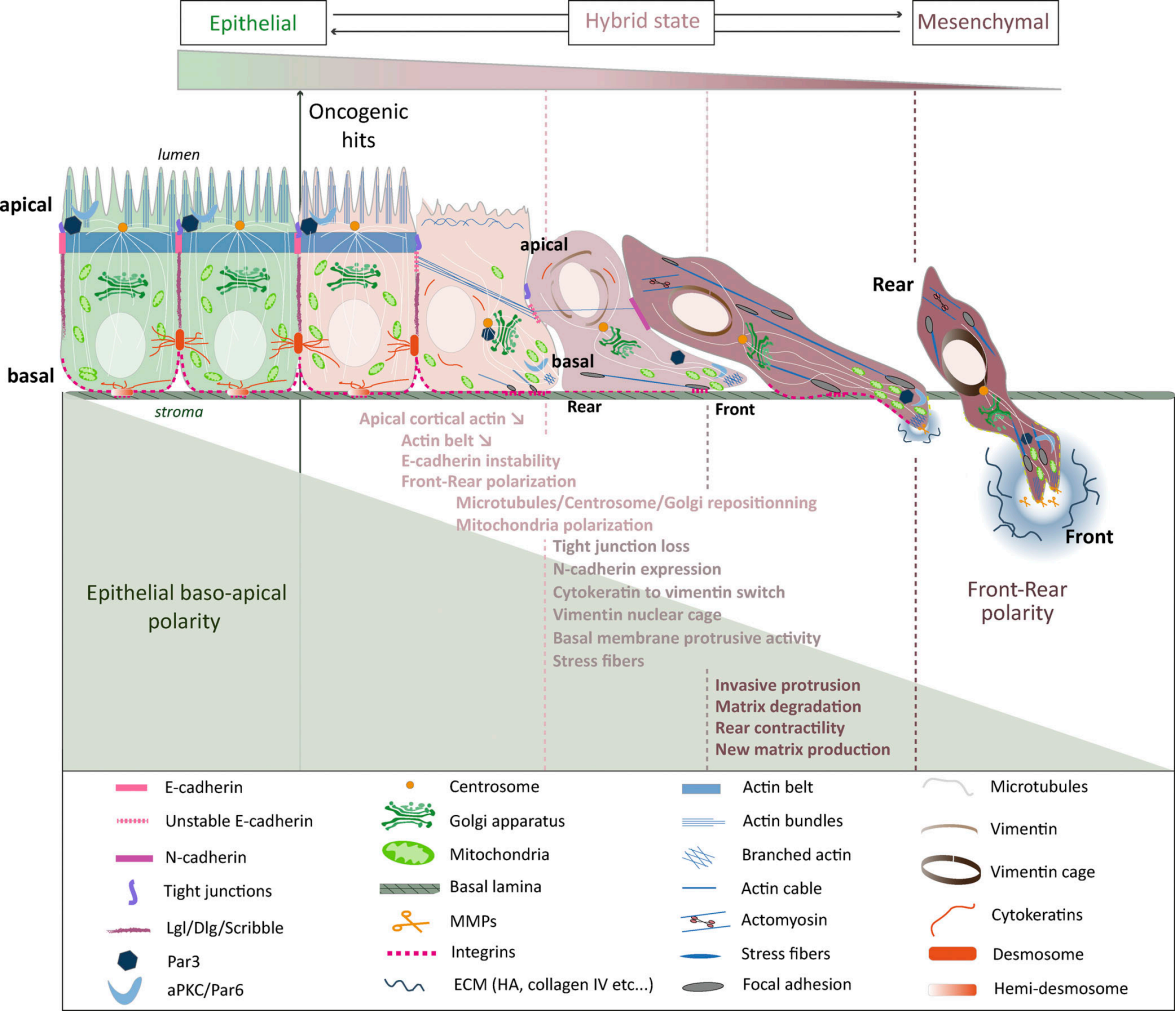
Basoapical to front-rear polarity conversion during epithelial-to-mesenchymal transition.

**Epithelial-to-mesenchymal transition (EMT):** Multicomponent process, mostly reversible, turning basoapical polarized epithelial cells into front-rear polarized invasive cells. The most frequent phenotypic changes involve progressive loss of cell–cell adhesion due to E-to-N-cadherin switch, tight junction loss, and desmosome disconnection, promigratory cytoskeleton network remodeling, and extracellular matrix degradation. Molecularly, the repression of the epithelial features and the production of mesenchymal-like proteins such as MMPs, vimentin, or N-cadherin is mostly triggered by the activation of a web of transcription factors (Snail, Slug, Twist, Zeb). See Fig. 2.

**Hybrid EMT:** Cells in hybrid EMT states express a mixture of epithelial and mesenchymal markers. This is usually the case for carcinoma cells invading collectively. Early partial and intermediate hybrid states correspond to the beginning and the middle of the EMT spectrum. For more information, the reader is referred to the guidelines written by the EMT International Association (Yang et al., 2020).

Redundancy in protein isoform functions limits our understanding of the role of these proteins in cancer development. For example, Lgl1 deletion in Nestin+ neural cell progenitors triggers brain malformation but not cancer (Jossin et al., 2017), whereas mice with Lgl2 null mutation do not show any obvious adult phenotype (Sripathy et al., 2011). However, a recent preprint revealed that joint Lgl1/Lgl2 deletion in keratinocytes sensitizes the epidermis to p53 hemizygous deletion and triggers squamous cell carcinoma formation (Bii et al., 2023 *Preprint*). Interestingly, total loss of Lgl leads to hyperproliferation of p53−/+ keratinocytes through aPKCι overactivation-dependent stimulation of the NF-kB signaling pathway.

### Changes in cell polarity promote tumor spreading

A key step in tumor cell transformation is the acquisition of migratory properties. Since cell migration requires a front-rear polarity axis fundamentally different from the basoapical polarity axis, initiation of migration is linked to an alteration of epithelial polarity. Tumor cells can undergo a full epithelial-to-mesenchymal transition (EMT), resulting in individual cell dispersion (Fig. 2 and glossary text box). However, a key question that remains unanswered to date is whether the disruption of epithelial polarity can initiate EMT or if it is solely a consequence of EMT.

**Figure 3.**
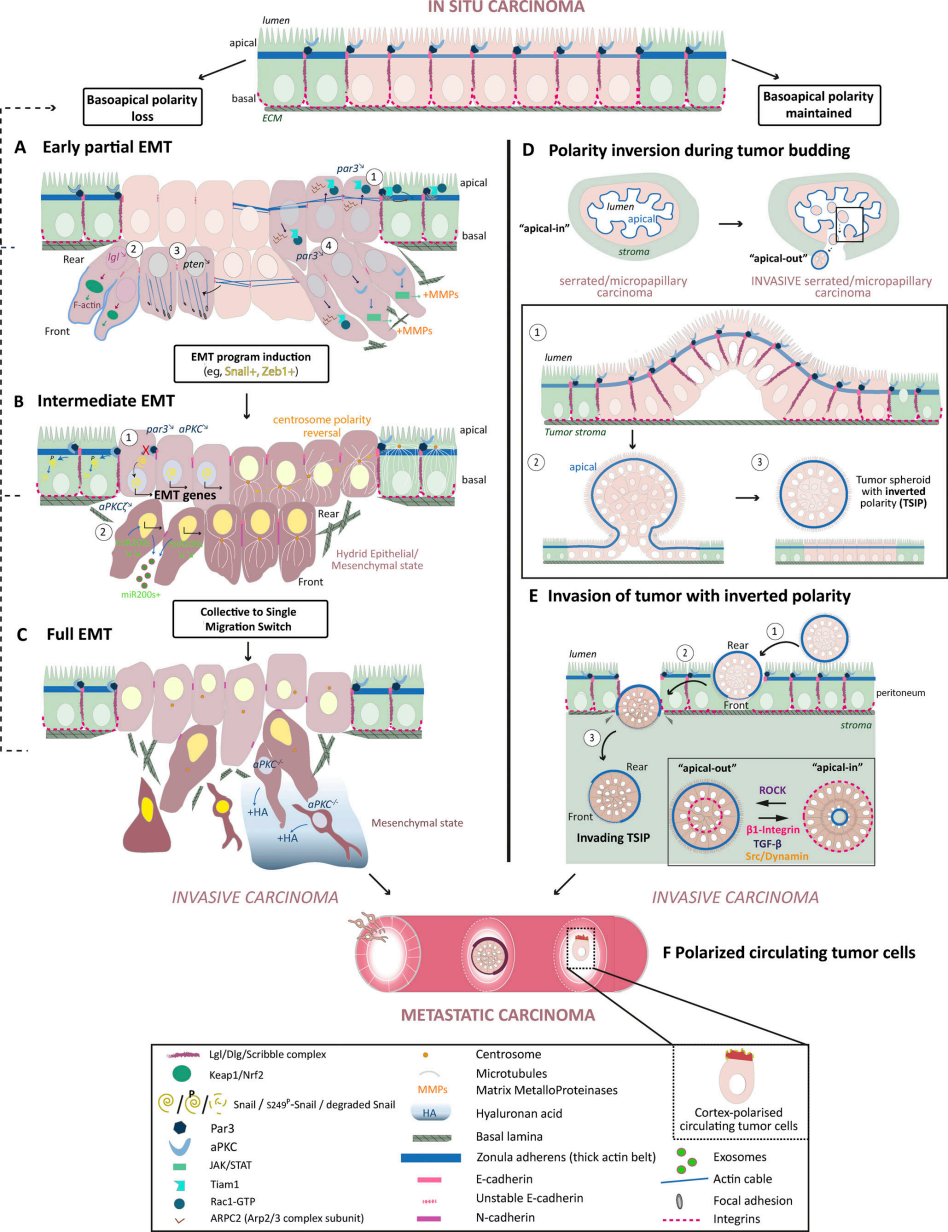
**Basoapical polarity changes enhance cancer-invasive properties.** Two main types of basoapical polarity changes turn in situ carcinomas into invasive and metastatic cancers: the progressive loss of basoapical polarity and the inversion of basoapical polarity. Progressive basoapical polarity loss is caused directly by cell polarity protein alterations or indirectly by disorganization of cell–cell and cell–ECM adhesions. Basoapical polarity loss is at the heart of the epithelial-to-mesenchymal transition. Progressive acquisition of mesenchymal traits turns stationary carcinoma cells into highly invasive cells (A–C). See Fig. 2 and text box for definitions of EMT. **(A)** Cell polarity protein alterations can trigger carcinoma invasion without fully activating EMT transcription programs. Because the complete absence of mesenchymal traits can’t be ruled out, this type of phenotypic changes could be at the very beginning of the EMT spectrum and is thus called early partial EMT. The cancer cells retain intercellular connections and epithelial traits but remodel their actin network to promote cell migration and weaken cell–cell adhesion (A1–A3). Molecular alterations include downregulation of Par3 expression leading to mislocalization of Rac1 activity and the Arp2/3 complex resulting in loosened actin belt at the zonula adherens (A1). Lgl and PTEN knockdown both promote collective cancer invasion by affecting the actin cytoskeleton (A2 and A3). Finally, par3 downregulation could also favor collective invasion by promoting MMPs’ secretion and ECM degradation, two traits usually associated with mesenchymal cells (A4). **(B)** Cell polarity protein alterations such as Par3 and aPKC downregulation can directly cause activation of EMT transcription by uplifting their inhibitory targeting of the EMT transcription factor Snail1 (B1). aPKCζ depletion indirectly triggers EMT program by reducing the intracellular level of miR200s via promotion of its secretion into exosomes, which activates Zeb1 expression, another EMT transcription factor (B2). Intermediate acquisition of the mesenchymal phenotype leads to an epithelial/mesenchymal hybrid state, which favors collective carcinoma invasion as cells retain strong enough intercellular adhesions. **(C)** Full epithelial-to-mesenchymal conversion and switch toward single cancer cell invasion is often observed in advanced metastatic cancer such as mesenchymal colorectal cancer. The absence of both aPKC kinases in intestinal stem cells is sufficient to trigger such cancers, whose invasive phenotype is promoted by the secretion of the ECM molecule hyaluronan acid (HA). **(D)** Carcinoma cells with intact basoapical polarity can collectively invade the tumor stroma after forming a bud which detach from the tumor core. This is typically observed in the micropapillary carcinoma and the serrated colorectal carcinoma. In the latter case, abnormal cancerous epithelium of intestinal glands bud apically within the lumen. As a result of this phenomenon, cancer spheroid bearing a supracellular actomyosin cortical belt on the outside detach and fill the lumen (D1–D3). The lumen of these glands eventually collapses which releases the spheroid in the stroma. **(E)** Once in the stroma, the spheroid contacts ECM molecules to repolarize their apical on the inside (“apical-in”). However, in some cases, this conversion does not occur leaving the tumor spheroid with an inverted basoapical polarity (TSIP). Interestingly these TSIP are able to invade the peritoneum, are highly metastatic and better resist chemotherapy than “apical-in” spheroids. The invasive process of the TSIP is just beginning to be understood (E1–E4). They likely perform a collective amoeboid mode of migration allowed by polarized supracellular actomyosin contractility at the rear of the spheroid coupled to the general jiggling of the cells, which generates friction forces necessary for forward movement (Pagès et al., 2022). **(F)** Finally, whereas clustering of individually invading mesenchymal carcinoma cells within the vasculature promotes metastasis success, single circulating tumor cells can increase their chance of secondary metastasis growth by polarizing their cortex to increase adhesion and extravasation.

EMT is associated with changes in the expression and activity of polarity proteins and with the remodeling of cell–cell junctions and cytoskeleton (Gandalovicova et al., 2016). Centrosome relocalization at the front of the nucleus is required for front-rear polarization and cell dispersion (Burute et al., 2017). Polarity complexes involving Cdc42, Par6, aPKC, Dlg, and Scrib localize at the front side of the migrating cells to control microtubule-dependent centrosome repositioning (Etienne-Manneville and Hall, 2001, 2003; Etienne-Manneville et al., 2005; Osmani et al., 2006). When cells are still interacting via cadherin-mediated adherens junctions, these contacts control the localization of polarity proteins and therefore control centrosome positioning (Dupin et al., 2009). Cell–cell adhesion defects during early EMT-primed cancer cells are thus likely to disrupt basoapical polarity. They may also promote the front-rear axis to ensure dissemination independently of any genetic alteration of polarity genes. However, a recent study revealed that, in fully polarized mammary epithelial organoids, aPKC, in complex with Par3, safeguards epithelial integrity by phosphorylating pro-EMT transcription Snail1 and targeting it for degradation (Jung et al., 2019). In this case, Par3 or aPKCζ+ι deletion is needed to trigger Snail1-induced EMT, cell invasion in vitro*,* and tumor metastasis in vivo (Jung et al., 2019). aPKC alteration can also promote EMT by affecting the level of EMT-inhibiting intracellular miRNA. In colorectal adenocarcinoma cells, the depletion of aPKCζ triggers the extracellular release of miR200s in exosomes which reduces miR200s intracellular level and causes pro-EMT molecular changes (Shelton et al., 2018; Fig. 2 and Fig. 3 B). These observations suggest that polarity alterations are required for EMT and tumor progression, although the full extent of the mechanisms at play still needs to be explored.

Instead of a full transition to the mesenchymal state, which requires the loss of cell–cell contacts, invading cancer cells can transition to hybrid states and migrate in a collective manner (Pastushenko et al., 2021; Fig. 2 and glossary text box). Par3, which localizes at cell–cell contacts in cell collectives, appears as a key element to control this invasive behavior. Remarkably, Par3 expression is lower in metastases compared with primary matched human breast tumor samples, suggesting that Par3 is key to preventing breast cancer from metastasizing (Xue et al., 2013). Groundbreaking studies by the Macara and Muthuswamy labs revealed that Par3 downregulation promotes invasion and metastasis in two different in vivo breast cancer transplant models, without inducing a typical EMT transcription program (McCaffrey et al., 2012; Xue et al., 2013). Xue et al. (2013) showed that in ErbB2+ breast cancer cells, loss of Par3 releases the Rac1 guanosine exchange factor (GEF) Tiam1 from junctions and activates Rac1 in the cytosol. The Arp2/3 complex is then localized away from cell–cell junctions, reducing cortical actin at the zonula adherens and modifying cadherin dynamics (Fig. 3 A). In squamous cell carcinoma, Par3 is also essential at cell–cell contacts. However, in this case, it recruits RhoE to antagonize ROCK-mediated contractility within the cell cluster and favors supracellular transmission of forces at the cell-free periphery of the cluster to enhance cluster migration and invasion (Hidalgo-Carcedo et al., 2011).

Par3 is not the only polarity protein capable of regulating actin rearrangements to affect cancer cell invasive properties. A recent study also revealed that alteration of the polarity gene and tumor suppressor *pten* leads to the disruption of the adherens junction and to the formation of focal adhesion-associated stress fibers, which boost glioblastoma cell migration (Peglion et al., 2022). The loss of PTEN activity induces an increased phosphorylation and activation of the AMP-activated protein kinase (AMPK) which controls the assembly of actin cables via Vasp. Inactivation of AMPK in PTEN-defective cells blocks cell invasion in vitro (Peglion et al., 2022; Fig. 2 A). Moreover, single-cell Omics analysis in a *Drosophila* model of ovarian cancer recently revealed that loss of basoapical polarity in Lgl knock-down follicle cells triggers collective invasion (Chatterjee et al., 2022) by increasing the polarization of actin in leader cells. While the complete loss of junctions can trigger the change of polarity required for single-cell invasion, alteration of polarity signaling may initiate a partial EMT by affecting cell–cell junction dynamics and signaling, and possibly facilitate the full EMT. The role of polarity proteins in the control of actin dynamics appears a key element in this event; however, the contribution of the regulation of the microtubule network, also probably involved, remains to be elucidated.

Cell migration evidently promotes tumor spreading in the surrounding tissues. However, the role of EMT in cancer metastasis remains debated, as recently illustrated in multiple reviews (Aiello and Kang, 2019; Bornes et al., 2021; Mittal, 2018; Williams et al., 2019). Cancer cells could migrate while keeping their basoapical polarity intact. In invasive ductal breast carcinoma, the integrity of adherens junctions is key to permitting both efficient collective invasion and metastatic secondary growth (Padmanaban et al., 2019). In hypermethylated colorectal cancers, metastasis into the peritoneal cavity involves the E-cadherin-dependent formation of tumor spheroids with inverted polarity (Zajac et al., 2018). The inversion of the apical and basolateral poles generates cancer clusters without adhesion on their periphery (Okuyama et al., 2016; Pagès et al., 2022; Zajac et al., 2018; Fig. 3 D). However, this polarity inversion has not yet been associated with a molecular alteration of polarity proteins. In fact, it seems that polarity signaling is modified so that in a confined environment, cancer clusters can develop a multicellular front-rear polarity axis to migrate collectively in a traction-force independent mode (Pagès et al., 2022; Fig. 3 E). Elucidating how cells maintain their basoapical polarity while creating a superimposed supracellular front-rear polarity will likely reveal novel insights into the importance of cell polarity changes in driving cancer dissemination (Capuana et al., 2020).

The interactions between cancer cells are also important during the passive dissemination of cell clusters through the circulation, as they must resist fluid shear stress (Huang et al., 2018) and immune attacks (Mohme et al., 2017). Clustered circulating tumor cells (CTCs) have been shown to be 20- to 100-fold more efficient at seeding metastasis in patient-derived breast cancer models (Aceto et al., 2014; Liu et al., 2019b). Interestingly, single CTCs incapable of regrouping can increase their seeding potential by generating a specialized cortical polarity axis. Liquid biopsies in patients with various cancers revealed the presence of CTCs with a cortical pole enriched in PI(4,5)P2 membrane folds, actomyosin, Ezrin, and adhesion molecules (Lorentzen et al., 2018). Depolarizing the cells through both genetic and non-genetic approaches reduces attachment, transmigration, and lung metastasis formation in a mouse model. In vitro analysis revealed that polarized CTCs do not migrate faster but better adhere to the endothelium, suggesting that cortical polarity of single CTCs promotes circulation arrest and extravasation (Lorentzen et al., 2018).

The interplay between polarity signaling and extracellular matrix (ECM) remodeling is another important way by which polarity proteins influence cancer progression (Winkler et al., 2020; see glossary text box). Activation of EMT transcription program triggers ECM remodeling (Eckert et al., 2011; Leong et al., 2014; Yeung and Yang, 2017). Conversely, the EMT-driven matrix deformation further reinforces basoapical polarity alterations, causing a deleterious feedback loop (Tharp and Weaver, 2018). Intriguingly, the alteration of polarity proteins can directly affect the ECM through the control of cell secretion. In invasive breast cancers, Par3 deletion activates aPKC and its downstream JAK/Stat3 pathway to promote the production of matrix-metalloproteinase enzymes (MMPs). Increased degradation of the ECM promotes the detachment of Par3-deleted cancer cell clusters and metastasis (McCaffrey et al., 2012; Fig. 3 A).

Polarity proteins can also influence ECM remodeling by controlling the expression of ECM components. A double depletion of aPKCι and aPKCζ in intestinal stem cells triggers aggressive mesenchymal colorectal tumors (mCRC) with a reactive desmoplastic environment (Nakanishi et al., 2018). Investigation of a large cohort of CRC specimens revealed that low levels of aPKC correlate with hyaluronic acid (HA) stromal deposition. Patients with low aPKC+high HA values had the worse prognosis (Martinez-Ordonez et al., 2022; Fig. 3 C). HA controls endothelial remodeling, cancer-associated fibroblast phenotype, and immune cell infiltration. Mouse models revealed aPKC depletion causes the overexpression of HA synthase, and drug treatment that degrades HA restores a less permissive tumor (Martinez-Ordonez et al., 2022). Whether aPKC controls HA synthesis and secretion through its cell polarity function remains to be explored.

### Alteration of polarity proteins promotes tumor resistance to metabolic stress, chemotherapy, and immune clearance

Changes in polarity protein expression can also promote cancer aggressiveness in more indirect and unexpected ways. Compelling evidence demonstrates the role of polarity proteins in cell adaptation to metabolic stresses. Deprivation of glucose typically reduces anabolism and cell proliferation. However, in colorectal cell lines, loss of aPKCζ increases the use of glutamine to maintain the energy production necessary for tumor growth (Ma et al., 2013). In Lgr5+ intestinal stem cells, whose proliferation is usually inhibited in nutrient-deprived situations, loss of aPKCζ reactivates cell proliferation and promotes tumorigenesis (Llado et al., 2015). In estrogen-receptor positive (ER+) breast cancer, Lgl2 recruits and stabilizes the amino acid transporter SLC7A5 at the plasma membrane. This increases the leucine uptake necessary for tumor cell proliferation under nutrient stress. High expression of Lgl2 and SLC7A5 correlates with poor survival in ER+ breast cancer patients treated with Tamoxifen, and depleting these proteins restored Tamoxifen sensitivity (Saito et al., 2019). Upon estrogen induction, Scrib level increases and Scrib recruits another amino acid transporter, SCL7A3, to form a quaternary complex with SCL7A5 and LGL2 and promote resistance to Tamoxifen in ER+ breast cancer (Saito et al., 2022). These observations suggest that the alteration of polarity proteins can participate in tumor cell metabolic adaptation. This is also illustrated by the regulation of the master metabolic player AMPK by several polarity proteins. The Par4/LKB1 protein, loss of which causes Peutz-Jeghers syndrome (PJS) characterized by hamartomatous polyps, directly phosphorylates and activates AMPK (Hardie and Alessi, 2013). By reducing AMPK activity, LKB1 alteration drives the metabolic reprogramming known as the Warburg effect, boosting glucose uptake and aerobic glycolysis to fuel many cancer aggressiveness (Bourouh and Marignani, 2022). However, in different settings, AMPK is overactivated following oncogenic alteration such as the loss of function of cell polarity gene PTEN in glial and glioblastoma cells (Chhipa et al., 2018; Peglion et al., 2022). In this aggressive brain tumor, overactivated AMPK drives a protumorigenic CREB-1-dependent bioenergetic network (Chhipa et al., 2018). Altogether, these studies reveal that context-specific metabolic reprogramming under nutrient stress is a crucial mechanism through which alteration of polarity proteins can contribute to cancer cell resistance. It would be interesting to determine if changes in cell metabolism observed following alteration of polarity genes also contributes to fueling tumor cell invasion (Garde and Sherwood, 2021; Parlani et al., 2023).

Cell polarity alterations also influence tumor cell responses to chemotherapy. The mislocalization of Scrib in lung adenocarcinoma cell lines induces adaptive resistance to kRAS-G12C inhibitors both in in vitro and in vivo xenografted mouse models (Adachi et al., 2023). kRAS-G12C inhibitors treatment reduces the expression of the palmitoyl S-acyltransferase ZDHHC7 by inhibiting the MAPK pathway. Altering palmitoylation relocalizes Scrib to the cytoplasm. Cytoplasmic Scrib promotes YAP nuclear translocation which induces MRAS expression and MRAS-dependent reactivation of the MAPK signaling pathway and increases cell resistance to treatment (Adachi et al., 2023). Previous studies in ER+ breast cancer tissues had already suggested that alteration of the Scrib complex proteins Lgl2, Scrib, and Dlg5 could boost Tamoxifen resistance (Liu et al., 2019a; Saito et al., 2019). However, depending on the treatment targets, the influence of polarity protein can vary. For instance, Scrib expression promotes the sensitivity of non-small cell lung carcinoma (NSCLC) to cisplatin, an inducer of reactive oxygen species (ROS) production (Wang et al., 2019). Downregulation of Scrib leads to the degradation of Nox2, a NAPDH oxidase that produces ROS and increases the tumor cell resistance. Cisplatin-resistant cancer cells express higher levels of PD-L1 which can be targeted by anti-PD-L1 therapy. Interestingly, the study also revealed that Scrib overexpression decreases PD-L1 levels in cisplatin-treated cells in vitro and in lung cancer models in vivo (Wang et al., 2019). Other examples illustrate the role of polarity protein in tumor cell sensitivity to treatment. For instance, Par3 expression further increases microtubule stabilization upon paclitaxel treatment favoring cell cycle arrest and apoptosis (Zhao et al., 2022). This may explain why the expression level of Par3 in breast cancer specimens correlates with paclitaxel treatment efficiency. Overactivation of aPKCι participates in the smoothened-inhibitor, vismodegib, resistance by bypassing Smo-dependent activation of HH signaling in basal cell carcinomas (Atwood et al., 2013). Finally, recent observations suggest that polarity proteins could play a role during chemotherapy-induced DNA damage responses (Shimada et al., 2022). Decreased aPKCζ expression in dedifferentiated chondrosarcoma is associated with a poor prognosis. Re-expression of aPKCζ activates the DNA-integrity checkpoint pathway ATM/chk2 and apoptosis, leading to a strong reduction of the tumor volume (Shimada et al., 2022).

In these examples, the contribution of polarity alteration to the effect on tumor resistance to chemotherapy is unclear for most studies. Instead, the role of polarity proteins in the control of polarity-independent signaling cascades is likely involved. However, the impact of changes in cell polarity on the tumor cell’s ability to resist chemotherapy has been highlighted in a few studies. Cell polarity inversion in primary colorectal cancer organoids is associated with increased chemoresistance (Canet-Jourdan et al., 2022), potentially via reversing the localization of multidrug resistance protein ABCB1 drug exporter on the outside of the tumor cluster, readily exposed to chemicals (Ashley et al., 2019). Also, loss of basoapical polarity favors resistance to Trastuzumab in HER2+ breast cancer cells by permitting MUC1-C interaction with abnormally localized HER2+, which constitutively activates the HER2 pathway and counteracts trastuzumab effect (Raina et al., 2014). Whether targeting polarity signaling may influence tumor sensitivity remains to be explored.

**Figure 4:**
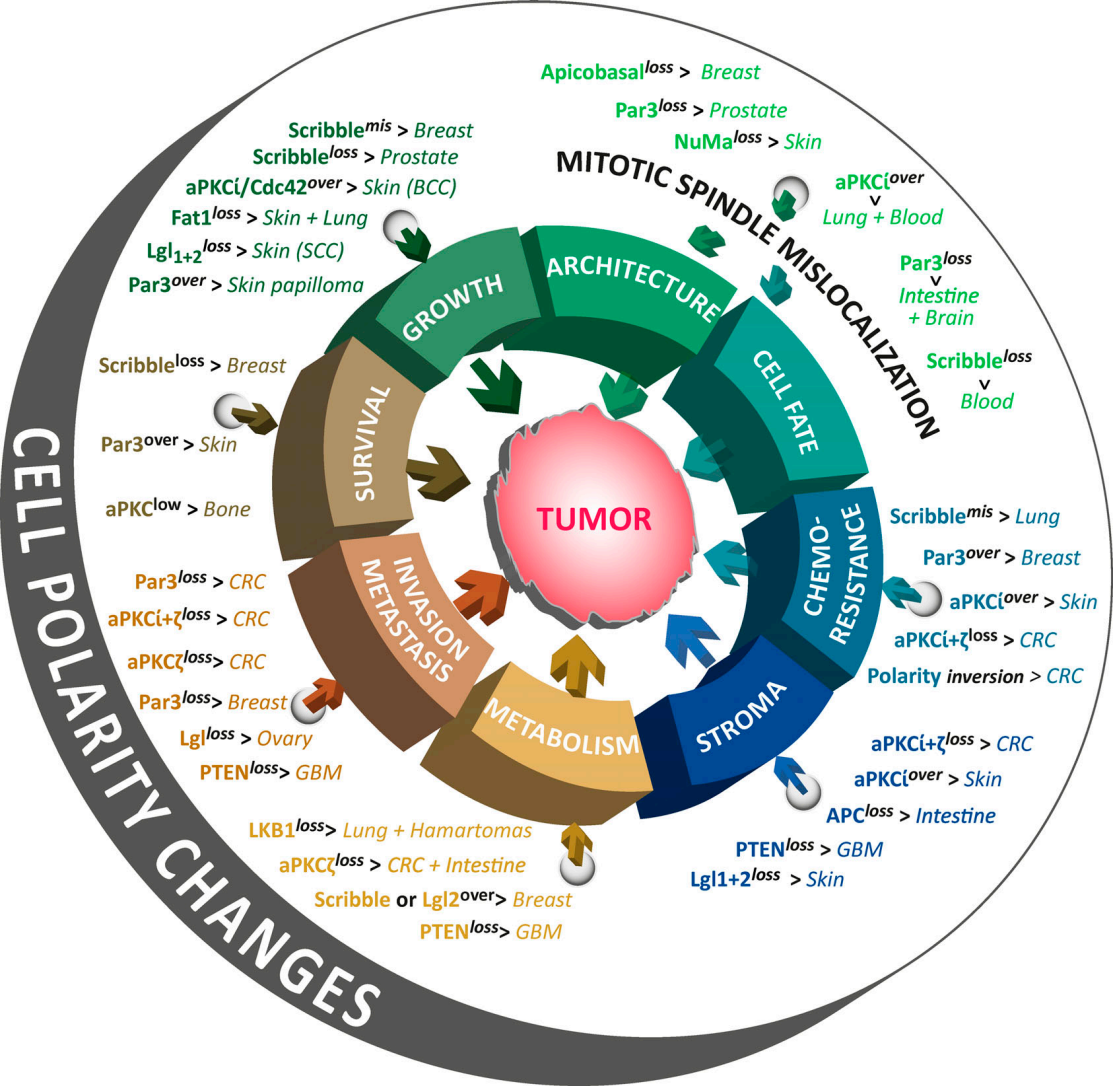
**Effects of altered cell polarity on cancer.** Major cell polarity protein alterations leads to cancer initiation and progression in their respective organs. The figure highlights the different biological processes affected by the cell polarity alterations that directly fuel tumor formation and aggressive behavior. The processes include cellular growth, cell survival, tissue morphogenesis, cell fate, cell invasion and metastasis, cell metabolism, tumor microenvironment (stroma) identity, and resistance to both chemotherapy and immune clearance. Polarity protein Adenomatous Polyposis Coli (APC) alteration in immune T cells is not mentioned in the text but its consequence on pro-oncogenic stroma alteration has been added in this figure (Mastrogiovanni et al., 2022a, 2022b).

Finally, and more surprisingly, alteration of polarity proteins has been suggested to contribute to the immunosuppressive tumor microenvironment. Loss of aPKCι and aPKCζ in intestinal stem cells promotes serrated colorectal cancer formation by preventing CD8^+^ T cells from penetrating the tumors. It also stimulates the presence of immunosuppressive CD4^+^ regulatory T cells and PD-L1 expressing myeloid-derived suppressor cells (MDSCs; Martinez-Ordonez et al., 2022; Nakanishi et al., 2018). However, this effect may be tissue-specific. In lethal serous ovarian cancers, it is the overexpression of aPKCι that initiates immune escape. It triggers Yap1-dependent expression of proinflammatory cytokines such as TNFα, which increases MDSCs infiltration and strongly suppresses cytotoxic T-cell proliferation (Sarkar et al., 2017). Lastly, PTEN alteration in glioblastoma cells facilitates poor-prognosis infiltration of the tumor microenvironment by macrophages. YAP-1 enrichment in PTEN-depleted cells promotes the expression and secretion of Lysyl Oxidase (LOX) in the tumor microenvironment, which attracts macrophages (Chen et al., 2019; Fig. 4).

The functions of polarity proteins not only in cancer cell resistance to treatment but also in cancer cell environment are only starting to be uncovered. Evidence suggests that their effects are pleiotropic and probably tissue-specific. Additional studies are clearly needed to come to clear conclusions in this field.

## Conclusion

Despite important research efforts, the role of cell and tissue polarity alterations in cell transformation and cancer initiation remains an open question. Still, a consensus now exists on the permissive or aggravating role of polarity protein alteration in cancer initiation and progression. Nevertheless, experimental evidence in physiological models showing that alteration of polarity proteins contributes to cancer by altering cell and tissue polarity is still largely missing.

On one hand, the change of polarity axis per se alters the polarized distribution of membrane receptors, intracellular signaling, and membrane trafficking. These alterations can contribute to the disorganization of tumor tissue and promote proliferative and invasive properties of cancer cells. During cancer spreading, polarity proteins, normally involved in basoapical polarity, play a key role in controlling the front-rear polarization of migrating cells and cell clusters. This change in cell polarity is associated with a rewiring of the polarity signaling; polarity complexes that antagonize each other to maintain the apical and basolateral domain identity can act cooperatively in migrating cells. For instance, the basolateral polarity protein Scribble and Dlg colocalize with the apical polarity proteins Par6/aPKC at the leading edge of migrating cells to ensure microtubule network reorientation and efficient migration (Abedrabbo and Ravid, 2020; Dow et al., 2007; Etienne-Manneville et al., 2005; Osmani et al., 2006). Moreover, basoapical and front-rear polarity can in some cases coexist to promote cluster cell dissemination. In this case, cell and tissue specificity are likely essential determinants to the positive or negative role of polarity proteins.

On the other hand, polarity proteins often take part in intracellular signaling that can affect proliferation, survival, ECM remodeling, or metabolism and likely influence tumor growth independently of their role in cell polarity. This is particularly due to the pleiotropic functions of most polarity proteins like Scrib, Par3, or aPKC. As cancer progresses, disseminates, and possibly resists immune reactions and therapies, the positive or negative role of polarity proteins is more and more difficult to determine. It would be important to determine whether the seemingly unrelated cell polarity-independent functions are in fact dependent on cell polarity.

The active investigation of the role played by cell polarity alteration in cancer reviewed here also gave rise to numerous studies showing cell polarity changes could be useful in the clinics as biomarkers to help refine diagnosis and prognosis for better standard of care (Table 1). It is clear that the wide variety of potential effects induced by the alteration of polarity will have to be taken into account if we hope to use them as potential therapeutic targets.

**Table 1.** Polarity changes as biomarkers

**Polarity alteration**	**Function**	**Type of biomarker**	**Marker**	**Cancer**	**Reference**
non specific	diagnostic: Tumoral (vs healthy)	Tissue staining (Immunohistochemistry)	Global apico-basal polarity loss	Majority of carcinoma	Routinely used in clinics
Lgl2 alteration	diagnostic: higher grades	Tissue staining (Immunohistochemistry)	Lgl2 loss or mislocalization	PDAC (pancreas)	Lisovsky et al, 2010
High Par3	diagnostic: metasatasis extrahepatic	Tissue staining (Immunohistochemistry)	High Par3 expression	HCC (liver)	Jan et al, 2013
low aPKCζ	diagnostic: more invasive-metastatic	Titration in serum	miR200s+-exosomes (in low aPKCζ CRC)	CRC (colorectal)	Shelton et al, 2018
Mislocalisation of Par6	diagnostic: higher grade, more invasive	Tissue staining (Immunofluorescence)	Mislocalized Par6 in nucleus	Breast cancer	Catterall et al, 2020
Par6 at membrane	diagnostic: tumour type	Tissue staining (Immunofluorescence)	Par6 at membrane	Luminal subtype (Breast cancer)	Catterall et al, 2020
low Crumb3	Pronostic: lower OS	Tissue staining (Immunohistochemistry)	Weak Crumb3 staining	ccRCC (Kidney)	Mao et al, 2015
High Par3	Pronostic: lower OS	Tissue staining	High Par3 staining	HCC (liver)	Li et al, 2021
Polarity inversion	Pronostic: lower OS and resistance to treatment	Tissue staining + Automated Morphometry	Inverted polarity (Cytokeratin, Mucus, stroma)	Mucinous CRC	Canet-Jourdan et al, 2022
low aPKCζ	Pronostic: lower OS	Tissue staining	Low aPKCζ	CRC	Yeo et al, 2018
low Par6β	Pronostic: lower OS	Tissue staining	Low Par6β	CRC	Yeo et al, 2018
low aPKCι+ζ	Pronostic: lower OS	Tissue staining	Low aPKCι+ζ/ High HA	mesenchymal CRC	Martinez-Ordoñez et al, 2022
low Lgl2	Pronostic: higher OS and DFS	mRNA level (qPCR)	Low Lgl2/Low SLC7A5	All breast cancer	Hisada et al, 2022
low Lgl2	Pronostic: higher OS (better response to tamoxifen)	mRNA level (qPCR)	Low Lgl2/Low SLC7A6	ER+_breast cancer + tamoxifen	Hisada et al, 2022
High aPKCι	Pronostic: lower OS and RFS	mRNA level (qPCR)	High aPKCι	Breast cancer	Catterall et al, 2020
High Dlg1	Pronostic: lower OS and RFS	mRNA level (qPCR)	High Dlg1	Breast cancer	Catterall et al, 2020
Low Par6β	Pronostic: lower OS and RFS	mRNA level (qPCR)	Low Par6β	Breast cancer	Catterall et al, 2020

Biomarkers are quantifiable indicators of physio-pathological features used to (1) differentiate healthy from cancerous tissues, (2) identify the aggressiveness of a cancer and predict the future course of the disease or (3) predict pharmacological response to treatments. Useful biomarkers in oncology comprise genetic alterations, abnormal protein expression or localization, morphological pattern on tissue sections, presence of circulated tumor cells, and extracellular vesicles in the blood. This table presents the cell polarity changes considered as diagnostic and/or prognostic biomarkers. The list is non-exclusive as it mainly highlights the most recent findings. For more complete information, the reader can refer to Halaoui and McCaffrey (2015); and Lo et al. (2012). OS, Overall Survival; DFS, Disease free Survival; RFS, Relapse-Free Survival. References from the table: Lisovsky et al. (2010); Jan et al. (2013); Mao et al. (2015); Li et al. (2021); Yeo et al. (2018); and Hisada et al. (2022).
